# Is maternal diabetes associated with autism spectrum disorder and ADHD in the offspring? A short review

**DOI:** 10.3389/fcdhc.2026.1855251

**Published:** 2026-07-15

**Authors:** Salam Alshammaa, Bashair M. Mussa, Amena Sadiya, Salah Abusnana

**Affiliations:** 1 Basic Medical Science Department, College of Medicine, University of Sharjah, Sharjah, United Arab Emirates; 2 Clinical Science Department, College of Medicine, University of Sharjah, Sharjah, United Arab Emirates; 3 Diabetes and Endocrinology Department, University Hospital Sharjah, Sharjah, United Arab Emirates

**Keywords:** attention-deficit/hyperactivity disorder, autism spectrum disorder, gestational diabetes mellitus, maternal diabetes, neurodevelopmental disorders, offspring outcomes

## Abstract

Maternal diabetes, encompassing gestational diabetes mellitus and pre-existing type 1 and type 2 diabetes are associated with adverse perinatal outcomes. Emerging evidence suggests that maternal hyperglycemia and insulin resistance may be associated with long-term neurodevelopmental outcomes, particularly increasing the risk of autism spectrum disorder (ASD) and attention-deficit/hyperactivity disorder (ADHD). This review synthesizes current epidemiological and mechanistic evidence linking maternal diabetes with the incidence of ASD and ADHD in offspring. Large population-based cohort studies consistently demonstrate an elevated risk of ASD and ADHD among children exposed to maternal diabetes *in utero*, with risk magnitude varying according to diabetes type, timing of diagnosis, and treatment modality. ADHD appears to show a stronger and more consistent association, particularly in offspring of mothers with type 1 diabetes. Proposed biological mechanisms include hyperglycemia-induced oxidative stress, epigenetic modifications, hormonal dysregulation involving estrogen, progesterone, and androgens, and an increased incidence of preterm birth. Maternal factors such as obesity, advanced age, and fertility treatments further modulate risk. Despite accumulating evidence, heterogeneity across studies and residual confounding limit causal inference. Future prospective studies integrating detailed glycemic profiling, hormonal assessments, and neurodevelopmental phenotyping are required to elucidate underlying mechanisms and inform preventive strategies for pregnancies complicated by diabetes.

## 1 Introduction

Maternal diabetes refers to high blood glucose levels detected during pregnancy, including gestational diabetes (GDM; diabetes developed during pregnancy) or pre-existing type 1 diabetes mellitus (T1DM) or type 2 diabetes mellitus (T2DM). Maternal diabetes and associated insulin resistance have been linked to adverse perinatal outcomes, including macrosomia, preterm birth, and stillbirth (1). Emerging evidence also suggests a possible association between maternal diabetes and long-term neurodevelopmental outcomes in offspring, particularly autism spectrum disorder (ASD) and attention-deficit/hyperactivity disorder (ADHD) (2, 3). ASD is a heterogeneous neurodevelopmental condition characterized by deficits in social communication and restricted, repetitive behaviors, with symptom severity ranging across a spectrum (4).

ADHD is a neurodevelopmental disorder characterized by symptoms of inattention, hyperactivity, and impulsiveness (5). Neurodevelopmental disorders like Autism showed an increase in prevalence among different regions ranging from 4% to 32% increase in the recent decades which represents a growing concern for its burden on healthcare systems and the families of the patients with these disorders (6). Recent evidence suggests that early embryonic brain development may influence the risk of ASD and ADHD (4). This review aims to summarize current evidence linking maternal diabetes with incidence of neuropsychiatric outcomes in offspring, with a focus on ASD and ADHD.

## 2 Methods

This narrative review was conducted through a structured literature search of PubMed, Scopus, and Google Scholar databases. Keywords included “maternal diabetes”, “gestational diabetes”, “autism spectrum disorder”, “ADHD”, and “neurodevelopmental outcomes”. Studies published in English from 2014 to 2024 were included. The literature search was conducted for studies published between January 2014 and December 2024. The final search was updated in April 2026 to ensure inclusion of recently published studies. In addition, reference lists of included articles and relevant review papers were manually screened to identify additional eligible studies. Titles and abstracts were screened for relevance, followed by full-text review of eligible articles. Studies meeting inclusion criteria were selected based on relevance to maternal diabetes and neurodevelopmental outcomes (ASD and ADHD). Observational studies (cohort, case-control, and population-based studies), systematic reviews, and meta-analyses were considered. Exclusion criteria included non-human studies (unless mechanistic relevance), case reports, and studies without clear neurodevelopmental outcomes. Due to the narrative nature of this review, no formal risk-of-bias scoring or meta-analysis was performed; however, study design and limitations were critically considered during interpretation.

## 3 Discussion

### 3.1 Maternal diabetes and incidence of ASD and ADHD

Large population-based studies indicate that exposure to maternal diabetes during gestation has been associated with an increased risk of ASD (7). In a cohort study of over 322,000 children followed for a mean of 5.5 years, Xiang et al. reported the highest ASD risk among offspring of mothers with GDM, followed by those with pre-existing T2DM (7). Among children diagnosed with ASD, approximately 9% were exposed to GDM and 3% to maternal T2DM.

Beyond the overall association with ASD risk, some studies have examined whether maternal diabetes is linked to specific ASD phenotypes or subtypes. Supporting these findings, Kania et al. reported an increased prevalence of ASD among offspring of mothers with GDM, with Asperger’s syndrome being the most frequently diagnosed subtype (8, 15). Another study demonstrated that children of mothers who had GDM were at 3.14-fold higher risk for developing ASD. The study results indicated that association of GDM with ASD risk remained significant after adjustment for major confounding factors, including maternal age and obesity; however, the possibility of residual confounding cannot be excluded (9).

Children born to mothers with T1DM also demonstrate a modestly increased risk (2.4% vs 1.8%) of ASD compared to those born to healthy mothers with no diabetes (10). The authors have attributed this association partially to higher rates of preterm birth among women with T1DM, a known risk factor for neurodevelopmental disorders.

Evidence indicates an association between *in utero* exposure to maternal diabetes and an increased risk of ADHD in offspring. In a large population-based cohort study involving 333,182 children, of whom 37,878 were exposed to maternal diabetes, the incidence of ADHD was highest among offspring of mothers with T1DM, followed by T2DM and GDM, with reported rates of 9.2%, 6.2%, and 4.8%, respectively. A study by Namoura et al. reported lower attention levels among children exposed to GDM *in utero*, with most ADHD diagnoses occurring between 4 and 6 years of age, and a higher prevalence observed among Caucasian children (11).

### 3.2 Hyperglycemia management

The findings further suggested that antidiabetic treatment during pregnancy may influence neurodevelopmental outcomes, as children born to mothers with GDM who received pharmacological treatment exhibited a higher incidence of ADHD compared to those whose mothers were managed without hypoglycemic agents (12). A study on 200,000 plus deliveries in 2016, revealed a plausible association between higher incidence of autism in the offspring when GDM was managed with diet and lifestyle alone, compared to management of GDM with insulin or oral hypoglycemic agents (13). Another study from national registry of Finland revealed that highest rates of neuropsychiatric disorders including autism concluded that the risk was highest for those exposed to insulin-treated pregestational diabetes, followed by non-insulin-treated T2DM and GDM (14).

### 3.3 Hyperglycemia and epigenetic modifications

Maternal hyperglycemia has been hypothesized to be associated with increased oxidative stress and altered epigenetic regulation based largely on experimental and animal studies. Persistent suppression of antioxidant genes such as superoxide dismutase 2 (SOD2) has been observed in offspring exposed to hyperglycemia *in utero*, potentially contributing to neuronal vulnerability and autism-like behaviors (15–17).

### 3.4 Hormonal dysregulation

Altered estrogen signaling has been proposed to be involved in metabolic and neurodevelopmental pathways, although evidence in humans remains limited. Estrogen receptors (ERα and ERβ) play critical roles in glucose homeostasis, inflammation regulation, and neurogenesis (18–21). Experimental evidence indicates that activation of estrogen signaling modulates neural development by reducing the proliferation of progenitor cells and regulating phenotypic outcomes associated with gene disruptions implicated in ASD, thereby suggesting a potential neuroprotective role of physiological estrogen signaling against ASD-related neurodevelopmental alterations (21). In contrast, the relationship between prenatal estrogen exposure and the development of ADHD remains insufficiently characterized. Nevertheless, emerging evidence suggests a potential association, as ADHD symptoms have been reported to fluctuate across the menstrual cycle in affected females (22). Additionally, reduced serum levels of G-protein–coupled estrogen receptor, a key component of estrogen signaling pathways, have been observed in children with ADHD, further supporting a possible link between disrupted estrogen signaling and ADHD pathophysiology (23).

It is reported that progesterone increases gluconeogenesis in the liver, in contrast, it has a homeostatic effect on the pancreas, where induces insulin secretion, suggesting that in cases of retarded insulin action or insulin resistance like maternal or GDM, progesterone alters the homeostatic state of glucose, contributing to hyperglycemia (24). In addition, it was found that prenatal exposure to higher levels of progesterone increases the risk of developing ASD in the offspring, which might link maternal diabetes with the increased possibility of having children with ASD later in life (25). However, no consistent results were found in patients with ADHD, indicating the need for further research to establish a link between ADHD and prenatal levels of progesterone (26).

Compared with ASD, mechanistic evidence linking maternal diabetes to ADHD remains less extensively characterized. Proposed pathways include dopamine dysregulation, altered executive function development, and stress-axis programming; however, most supporting evidence is indirect or derived from animal and neuroendocrine models. Further human studies are required to clarify these mechanisms.

While ASD has been more extensively studied in relation to mechanistic pathways, ADHD shows a more consistent epidemiological association, though its biological mechanisms remain less well defined.

### 3.5 Androgen exposure

Androgens play a physiological role in fetal sexual differentiation and contribute to the maintenance of pregnancy, with circulating levels naturally increasing during gestation (27). However, supraphysiological androgen concentrations have been reported in females with metabolic disorders, including diabetes, during pregnancy (28). In addition, Gunes et al., 2020 suggests that prenatal exposure to elevated androgen levels might increase the risk of neurodevelopmental disorders, including ASD and ADHD, in the offspring. Notably, both ASD and ADHD exhibit a marked male predominance, supporting a potential role of androgen-mediated mechanisms in disease susceptibility (29). However, direct evidence linking prenatal androgen exposure to ASD and ADHD in humans remains limited and largely inferential.

### 3.6 Preterm birth

Maternal diabetes significantly increases the risk of preterm birth (30, 31), which itself is strongly associated with ASD and ADHD. Children born preterm (<33 weeks) demonstrate higher rates of cognitive, behavioral, and attentional difficulties, suggesting an indirect pathway linking maternal diabetes to neurodevelopmental outcomes (32, 33).

### 3.7 Confounding risk factors

Evidence has indicated that the development of autism in children to mothers with diabetes is influenced by other factors that exaggerate the risk as well, where it was found that maternal age, birth weight, fertility treatments, and maternal obesity increase the odds of having a child with ASD among mothers who had diabetes during pregnancy (8, 13, 34).

### 3.8 Critical appraisal of evidence

Most of the evidence linking maternal diabetes with ASD and ADHD is derived from observational cohort and case-control studies. While these study designs are essential for investigating long-term developmental outcomes, they are inherently limited in their ability to establish causality due to residual and unmeasured confounding. Important confounding factors include maternal pre-pregnancy obesity, socioeconomic status, genetic susceptibility, maternal education, lifestyle factors, medication use during pregnancy, and perinatal complications, all of which may independently influence neurodevelopmental outcomes.

Cohort studies generally provide stronger temporal inference because exposure is assessed prior to outcome development; however, they remain vulnerable to unmeasured confounders and misclassification of exposure or outcome, particularly in registry-based datasets. In contrast, case-control studies are more susceptible to recall bias and selection bias, especially when exposure data rely on retrospective reporting or administrative coding.

In addition, variability in diagnostic criteria and ascertainment methods for ASD and ADHD across countries, healthcare systems, and study periods contribute to heterogeneity in reported effect sizes. Differences in follow-up duration and age at diagnosis further influence incidence estimates, particularly for ADHD, which may be underdiagnosed in early childhood.

Furthermore, most studies are unable to fully account for the combined influence of maternal metabolic status, glycemic control, and treatment modality, which may act as effect modifiers. Publication bias toward positive associations may also contribute to overestimation of effect sizes in the literature.

Overall, while consistent associations are observed across multiple studies, the evidence remains associated rather than causal, and findings should be interpreted with caution.

## 4 Limitations

This review is limited by the heterogeneity of included studies, variability in diagnostic definitions, and reliance on observational data. In addition, differences in adjustment for confounders such as maternal BMI, genetics, and socioeconomic status limit comparability across studies. Mechanistic interpretations are largely based on preclinical evidence and should be interpreted cautiously.

## 5 Conclusion

Current evidence supports an association between maternal diabetes and increased risk of ASD and ADHD in offspring, with a stronger and more consistent association observed for ADHD. The magnitude of risk varies by diabetes type, timing of diagnosis, glycemic control, and maternal factors such as obesity and age. Proposed mechanisms include hyperglycemia-induced oxidative stress, epigenetic alterations, hormonal dysregulation, and increased risk of preterm birth.

Despite growing evidence, causality remains uncertain, and findings are heterogeneous. Well-designed prospective studies with detailed phenotyping, glycemic profiling, and mechanistic exploration are needed to clarify these associations and inform preventive strategies in pregnancies complicated by diabetes.
